# Suicide by Pesticide (Phorate) Ingestion: Case Report and Review of Literature

**DOI:** 10.3390/toxics10050205

**Published:** 2022-04-21

**Authors:** Angela Simonelli, Anna Carfora, Pascale Basilicata, Bruno Liguori, Pasquale Mascolo, Fabio Policino, Massimo Niola, Carlo Pietro Campobasso

**Affiliations:** 1 Department of Advanced Biomedical Science, Legal Medicine Section, University of Naples “Federico II”, Via S. Pansini, 5, 80138 Napoli, Italy; angela.simonelli@unina.it (A.S.); fabio.policino@unina.it (F.P.); masniola@unina.it (M.N.); 2 Forensic Toxicology Unit, Department of Experimental Medicine, University of Campania “L. Vanvitelli”, Via L. Armanni, 5, 80138 Napoli, Italy; anna.carfora@unicampania.it (A.C.); carlopietro.campobasso@unicampania.it (C.P.C.); 3 Department of Experimental Medicine, Institute of Legal Medicine, University of Campania “L. Vanvitelli”, Via L. Armanni, 5, 80138 Napoli, Italy; bruno.liguori@gmail.com (B.L.); studiomedicolegalemascolo@gmail.com (P.M.)

**Keywords:** phorate, suicidal ingestion, pesticides

## Abstract

It has been estimated that approximately one in seven of all global suicides is due to pesticide self-poisoning, mostly in rural areas of developing countries. Organophosphorus (OP) compounds are a group of pesticides exerting their toxicological effects through non-reversible inhibition of the enzyme acetylcholinesterase (AChE). Among these compounds, phorate (thimet) is one of the most dangerous compounds, the use of which is restricted in many countries. A case of intentional suicide after phorate ingestion in a 24-year-old Bengali male is described. This is the second case of suicidal ingestion of phorate reported in the forensic literature, and the first presenting complete toxicological findings.

## 1. Introduction

Based on the Food and Agriculture Organization of the United Nations definition [1], pesticides can be broadly described as chemical products used for the control of unwanted animals, plants and microbes. Pesticides are often classified according to the pest organism, to which they are designed (rodenticides, insecticides, weedicides, fungicides, acaricide, et cetera), however, the most useful classification is based on their chemical composition: organochlorines; organophosphorus; carbamates; and pyrethrin and pyrethroids [2,3]. Organophosphate pesticides are the most common pesticides used in agriculture, however, they can have a negative impact on both the environment and human health [4,5]. Several ecological and health concerns have been raised on the recurrent usage of organophosphate pesticides. Epidemiological studies of pesticide poisoning indicate three main categories of circumstances, under which poisoning occurs: occupational accidents after occupational exposures; domestic accidents; and suicide due to intentional ingestion [6,7,8,9]. A literature review of mortality studies related to suicide by organophosphate pesticides has suggested that the wide availability of highly toxic pesticides may have a causal relationship to suicide [9].

According to the World Health Organization hanging, pesticide self-poisoning and use of fire arms are globally the most common methods of suicide [10]. A systematic review of world data for 2010–2014 estimated that around one in seven of global suicides were due to pesticide self-poisoning (approximately 110,000 deaths each year) [11]. Mostly these deaths occur among people living in rural areas of low-income and middle-income countries, especially in South Asia, South East Asia and China [11,12].

Organophosphorus compounds (OP) are widely used as insecticides or acaricide and exert their toxicological effects through non-reversible inhibition of the acetylcholinesterase enzyme (AChE). Inhibition of AChE results in accumulation of acetylcholine (ACh) at autonomic postganglionic and central synapses and at neuromuscular junctions [13,14]. As a consequence, ACh binds to and over-stimulates muscarinic and nicotinic receptors. AChE inhibition is irreversible, therefore new synthesis of the enzyme in the liver is necessary to restore enzymatic activity [15].

Phorate is an OP compound available as a liquid or in granules. According to the WHO Recommended Classification of Pesticides by Hazard [10] it is classified as ‘extremely hazardous’ (class Ia-see Table 1), with an oral LD50 8.0 mg/kg mice, and 1.1–3.2 mg/kg to rats [15,16]. Although phorate is an extremely hazardous poison, only a few cases of fatal poisoning by phorate (both intentional and accidental) are reported in the literature [17,18,19]. This could be partially due to its limited use, since it is not approved for use in the EU [20]. The Environmental Protection Agency has authorized restrictions on its use in the US since 1990 [21]; in China, phorate and the metabolites of phorate sulfone are forbidden in food stuffs [16].

The present paper reports a case of suicide by phorate ingestion in a rural area of Southern Italy. To the best of our knowledge this is the second paper reporting toxicological data after suicidal phorate ingestion in Italy. Toxicological data and autopsy findings are discussed with respect to the literature data and recommendations for safety pesticides management.

## 2. Case Report

A 24-year-old Bengali male was found dead in a rural area of Southern Italy. The body was found on the ground in a supine position. Post-mortem changes were consistent with an early post-mortem interval of approximately 3–6 h, with no signs of putrefaction. At external examination, a marked miosis (pinpoint pupils) was observed along with a white foam coming out of the mouth and nostrils and semen leakage. Body weight was 56 kg and height 152 cm. At autopsy, performed according to a Prosecutor Office request, no evidence of traumatic injuries or relevant diseases were observed, except for an overdistension of the lungs with an overlap of their anterior edges over the midline.

Stomach contents were represented by 300 mL of an oily, sharp-smelling liquid containing pieces of a partially digested yellow citrus fruit. Hemorrhagic erosions of the gastric mucosa were observed along with diffuse visceral congestion; blood appeared fluid and dark red in color. According to the international recommendations on sample collection [22,23], 10 mL peripheral blood was taken from the right femoral vein. Urine, bile, and stomach contents were also sampled for toxicological analysis.

## 3. Materials and Methods

The certified standard solutions of drugs of abuse used for confirmation analysis in gas chromatography/mass spectrometry (GC/MS) were from Cerilliant-Merck (Milan, Italy), *N,O*-bis(trimethylsilyl)trifluoroacetamide (BSTFA) derivatizing agent from Acros (Morris Plains, NJ, USA), and HPLC grade-solvents from Carlo Erba (Milan, Italy).

Enzyme Linked ImmunoSorbent Assay (ELISA) screening tests were performed on a Dynex-DSX system from Technogenetics (Chantilly, VA, USA), using forensic blood kits from Abbott for AMP/MAMP/MDMA, barbiturates, benzodiazepines, buprenorphine, cannabinoids, cocaine, fentanyl, ketamine, methadone, opiates, oxycodone, tricyclic antidepressants, zolpidem. The immunoassay system is based on a heterogeneous assay with a fixed and constant antibody coating on the plate. All the antibodies are poly-clonal, except for amphetamine and methadone, which are mono-clonal ones. The enzyme conjugate contains a fixed titer of an enzyme (horseradish peroxidase) labelled drug. The immunoassay exploits a competitive binding between the antibody and the antigen.

GC/MS analyses were performed using a DSQII single quadrupole mass spectrometer directly linked to a FocusGC gas chromatograph equipped with a *full scan* AS3000 autosampler, all from ThermoFisher (San José, CA, USA). Gas chromatographic separations were performed with a DB-5ms Ultra Inert (30 m × 0.25 mm × 0.25 μm) (J and W Scientific, Folsom, CA, USA). Data were processed using the Xcalibur software (version 2.0.7) from ThermoFisher.

Head-space gas chromatographic/mass spectrometric (HS-GC/MS) analyses were performed on an HP6890 series gas chromatographer provided with a HP7694E autosampler and a 5973 single quadrupole mass spectrometer (Hewlett-Packard, Palo Alto, CA, USA); chromatographic separation was accomplished by a CP PorabondQ capillary column (Varian, Palo Alto, CA, USA), and data analyzed using the MSD Chemstation, with software (D.02.0.275 version) from Agilent Technologies.

### Toxicological Analysis

Biological fluids were used for complete toxicological analyses. ELISA screening tests were initially performed on a blood sample, after dilution (1:10, v:v) of a proper aliquot with bidistilled water, according to the manufacturer’s specifications.

Generic analyses were performed on blood, urine, bile and gastric contents, after purification through solid phase extraction (SPE) using BondElut Certified cartridges (Agilent Santa Clara, CA, US) with and without acidic hydrolysis [24,25,26]. Generic analysis with acidic hydrolysis. Sample preparation: 1 mL matrix diluted with 2 mL bidistilled water and added with 200 µL 37% HCl. Acidic hydrolysis: samples were kept at 120 °C for 20 min, then cooled at room temperature and added with 800 µL TRIS buffer and 200 µL 10 M KOH saturated with KHCO_3_ (samples’ pH 8–9). Samples were centrifuged at 4000 rpm for 40 min then purified. SPE purification procedure, involving a drop-to-drop elution at 5 mmHg, was performed as follows: conditioning, 1 mL methanol, 2 mL 0.1 M phosphate buffer (pH 6); sample loading; washing: 6 mL bidistilled water, 3 mL 0.1 N HCl; 9 mL methanol; cartridges were dried for 5 min, then clean test tubes loaded; elution: 2 × (3 mL dichloromethane/methanol = 80/20, 2% ammonia, v:v). Eluted fractions, dried under nitrogen stream, were derivatized by adding 50 µL BSTFA at 75 °C for 20 min; samples were cooled at room temperature and analyzed in GC/MS. Generic analysis without acidic hydrolysis. Sample preparation: 1 mL matrix diluted with 2 mL bidistilled water. Each sample was centrifuged at 4000 rpm for 40 min then purified. SPE purification procedure was performed as follows: conditioning, 1 mL ethyl acetate, 1 mL methanol, 1 mL bidistilled water, 1 mL 0.1 M phosphate buffer (pH 6); sample loading; washing: 3 mL bidistilled water, 3 mL 0.1 M phosphate buffer 3% acetonitrile; 3 hexane; cartridges were dried for 5 min, then clean test tubes loaded; elution: 2 × (3 mL ethylacetate, 2% ammonia, v:v). Eluted fractions, dried under a nitrogen stream, were derivatized by adding 50 µL BSTFA at 75 °C for 20 min; samples were cooled at room temperature and analyzed in GC/MS.

Toxicological analyses aimed to verify the presence of pesticides involved a liquid/liquid purification through TOXI-A and TOXI-B cartridges (Agilent, Santa Clara, CA, USA); solutions recovered by TOXI tubes were dried under nitrogen stream and re-dissolved in 50 µL acetonitrile prior to GC/MS analyses. Analyses were performed on fluids and gastric content; no hydrolysis nor derivatization were performed. Qualitative GC-MS *full scan* analyses were performed on all samples; selected ion monitoring mode (GC/MS-SIM) was used for quantifications of active principles, eventually evidenced by *full scan*. The eventual presence of ethyl alcohol or any other volatile chemicals in the blood sample were also verified by HS-GC/MS.

A five-point calibration curve in the range (0.016–0.250) mg/L was used for phorate quantification, using ethion as internal standard. GC/MS-SIM analyses were based on the following transitions: phorate, *m/z* (260, 231, 121); ethion, *m/z* (231, 153, 125); in both cases the first ion was selected as quantifier.

## 4. Results and Discussion

ELISA analyses were negative with respect to considered analytes. Generic analyses performed on samples purified by SPE with and without acidic hydrolysis were negative as well. No volatile compounds were evidenced in the blood sample by HS-GC/MS.

GC/MS *full scan* analyses on samples purified by TOXI-A and TOXI-B cartridges evidenced a positivity towards phorate. Quantification in blood, urine, bile and stomach contents involved GC/MS-SIM analyses of sample aliquots spiked with ethion (used as internal standard) and purified by TOXI-A. Specificity towards phorate identification was verified through the analysis of drug-free blood and urine samples spiked with the internal standard only and processed according to the previously described method. Results evidenced the absence of any signal corresponding to the analyte of interest. Limit Of Detection (LOD) and Lower Limit Of Quantification (LLOQ) were determined as concentrations corresponding to a signal-to-noise ratio of 3/1 and 5/1, respectively: LOD, 8.5 ng/mL; LLOQ, 14.1 ng/mL. Recovery and accuracy of the applied method were calculated for drug-free blood and urine spiked with phorate standard solution to obtain concentrations of 0.150, 0.075 and 0.037 mg/L (each sample was prepared in triplicate). After purification and GC/MS-SIM analysis, a mean recovery and an accuracy of 87% and 95% were obtained, respectively. Quantification involved the use of a calibration curve. Since to the best of our knowledge a toxic interval for human absorption was not available (in terms of expected blood concentrations), the calibration curve concentrations’ range was optimized to balance between the need of optimal ions’ intensities and to avoid the analysis of unnecessary excessively concentrated solutions—resulting in the need of frequent instrumental cleaning. Quantitative results are reported in Table 2 (bile and gastric content analyses were repeated after proper sample dilution with bidistilled water, in order to obtain concentration within the calibration curve range).

In samples purified by TOXI-A, GC/MS *full scan* analysis was able to detect the presence of phorate main metabolites like phorate sulphone and phorate sulphoxide. Both metabolites are reported to be more toxic than phorate [27]. The majority of toxicokinetic studies reported in the literature were based on laboratory animals, while data on humans are extremely limited [28].

Although a specific oral toxic or lethal dose for phorate in humans has not been established [29], the blood concentration of phorate found in this case study was very similar to the lethal concentration reported by Thompson et al. [30], and here considered as “reference” for assessing lethal phorate levels in humans. In describing the case of fatal ingestion of phorate-containing insecticide in a 17-year-old restaurant assistant, the authors reported a post-mortem blood insecticide concentration of 0.25 mg/L. In our case phorate blood concentration was close to the “lethal” concentration published by Thompson et al. [30]. The case discussed by Thompson et al. is one of the very few cases published in the literature where quantitative results of toxicological analyses are reported. Khatiwada et al. [18], for instance, published a case of accidental intoxication after ingestion of phorate granules with a fatal outcome in one of the two victims, however, results of toxicological analyses are not reported. Peter et al. [31] reported a non-fatal case of a 28-year-old woman who swallowed 50 mL of phorate. She survived after five days in a deep coma, during which findings largely consistent with brain death were observed.

In our case study high phorate concentration was also determined in the stomach content (11.52 mg/L). Considering the entire volume of matrix present at autoptic examination, an ingestion of at least 3.4 mg of phorate can be estimated. In a recent study, Montana and colleagues [19] published the case of a suicide following phorate ingestion by a 70-year-old farmer, who had been exposed for several years to phorate at low doses and died after ingestion of a granular pesticide powder mixed with water. Reported toxicological datum refers to gastric content only and phorate levels of 3.29 mg/L were determined. In our case, the main histological findings were represented by brain and pulmonary oedema associated to alveolar hemorrhages consistent with an acute fatal event. Similar histological findings have been also observed by Montana et al. [19], consistent with the acute toxicological effects of OP poisoning on the nervous and respiratory systems along with additional signs of chronic obstructive pulmonary disease, such as scattered bronchopneumonia outbreaks and peribronchiolar lymphocytes infiltrations and alterations of kidney due to chronic exposure to OP.

In our case toxicological findings, along with those derived from the autopsy, were consistent with an acute intoxication due to phorate intentional ingestion. Among others, the pungent smell typical of pesticides coming from the stomach contents, signs of overstimulation of the parasympathetic nervous system (miosis, white foam that comes out of the mouth and nostrils, ejaculation of the semen), were found along with the exclusion of traumatic lesions or relevant underlying diseases as possible causes of death. Miosis has been found to be one of the most prevalent signs in insecticide poisoning in a range between 44–83% of cases [32,33]. Miosis and tightness in the chest may occur as the result of the severity of local anti-AChE effects [13]. Such autopsy findings are consistent with the lethal effects of OP insecticide poisoning. These compounds can produce a variety of toxicological effects on the nervous (central and peripheral) system, as well as cardiovascular and pulmonary systems such as: ventricular arrhythmias and tachyarrhythmias belonging to the ‘torsades de pointes’ types, which may progress to ventricular fibrillation and/or asystole; twitching of muscles followed by convulsions and paralysis with respiratory failure associated to nasal and bronchial hypersecretion (rhinorrhea and bronchorrhea) as a result of bronchoconstriction, cyanosis and respiratory depression [13]. In the case presented here both circumstantial and toxicological findings were in agreement and provided evidence to confirm the diagnosis of OP poisoning.

With respect to the particular absorption route, OP pesticides can efficiently enter the organisms by all routes, including inhalation, ingestion, and dermal absorption [14]. The latter two routes of absorption concern mostly agricultural workers, accidentally exposed either during pesticide application to crops or due to incorrect or careless storage. Symptoms may occur within five minutes of massive ingestion and almost always within twelve hours [13]. Work-related poisoning can be ruled out in the case discussed here due to the high concentration present in the gastric contents and signs evidenced during the autopsy, which were consistent with an ingestion of the toxicant.

In cases of acute OP poisoning, antidotal treatment with the combined use of atropine sulfate and pyridine-2-aldoxine methochloride (2-PAM) is recommended. Atropine sulfate acts by blocking the muscarinic Ach receptors (mAChRs) [15]. In case of ingestion, gastric lavage can be considered especially in the first hours after poisoning occurs, although its value is unproven [34], also in consideration of the potential harm it can cause if the patient, at risk of seizures or rapid loss of consciousness, has not been intubated [35]. Ventilation must be maintained in patients with acute OP poisoning; eventually patients must be intubated and sustained by mechanical ventilation [13]. Finally, careful attention must be given to fluid and electrolyte balance along with heart rate and blood pressure monitoring by ECG. Unfortunately, there are conflicting recommendations on the general management of poisoned patients and the lack of evidence for the treatment of acute OP poisoning has been raised [36,37,38].

## 5. Conclusions

Pesticide self-poisoning is very common in rural areas of developing countries. Unfortunately, few details are available about phorate toxicity on humans. Further studies dealing with toxicokinetic and toxicodynamic of OP compounds are still needed. According to the autopsy findings, the effects of OP poisoning can determine a rapid death due to brain and respiratory alterations through non-reversible inhibition of the enzyme AChE.

## Figures and Tables

**Table 1:** World Health Organization classification for pesticides toxicity.

WHO Class		LD50 for the Rat (mg/kg Body Weight)	
		Oral	Dermal
Ia	Extremely hazardous	<5	<50
Ib	Highly hazardous	5–50	50–200
II	Moderately hazardous	50–2000	200–2000
III	Slightly hazardous	Over 2000	Over 2000
U	Unlikely to present acute hazard	5000 or higher	

**Table 2:** Quantitative results of phorate in analyzed biological samples.

Samples	Phorate (mg/L)
Blood	0.18
Urine	0.01
Bile	1.12
Stomach contents	11.52

## Data Availability

Not applicable.
